# Supplementary material for: Respiratory virus circulation during the first year of the COVID‐19 pandemic in the Household Influenza Vaccine Evaluation (HIVE) cohort
Source: Influenza Other Respir Viruses. 2023 Mar 1;17(3):e13106. doi: 10.1111/irv.13106 (PMC9975790; doi:10.1111/irv.13106)
Supplement: Supplementary file 1 — Table S1. Coinfections detected among HIVE study participants. [file IRV-17-e13106-s001.docx]

**Table S1. Coinfections detected among HIVE study participants.**

| **Coinfection** | **Count (%) n=23** |
| --- | --- |
| RV, ADV | 6 (26.1) |
| RV, HMPV | 2 (8.7) |
| RV, HBoV | 2 (8.7) |
| RV, SARS-CoV-2 | 3 (13.0) |
| RV, CoV 229E | 1 (4.3) |
| RV, CoV OC43 | 1 (4.3) |
| CoV NL63, EV | 1 (4.3) |
| CoV NL63, SARS-CoV-2 | 1 (4.3) |
| ADV, HBoV | 1 (4.3) |
| Influenza A, Influenza B | 1 (4.3) |
| Influenza A, RSV | 1 (4.3) |
| Influenza A, CoV 229E | 1 (4.3) |
| HPIV-3, HBoV | 1 (4.3) |
| RV, ADV, HBoV | 1 (4.3) |

Abbreviations: AdV, adenovirus; CoV, coronavirus; EV, enterovirus; HBoV, human bocavirus; HMPV, human metapneumovirus; HPIV, parainfluenza virus; RSV, respiratory syncytial virus.
